# Current status and future directions of *Chlamydia pecorum* research: a scoping review (2010–2025)

**DOI:** 10.3389/fvets.2026.1799591

**Published:** 2026-04-01

**Authors:** Huong Q. Duong, Jessie S. F. Wong, Melinda Doyle, Samuel Phillips, Nina Pollak, Peter Timms, Martina Jelocnik

**Affiliations:** 1 Centre for Bioinnovation, University of the Sunshine Coast, Sunshine Coast, QLD, Australia; 2 School of Science, Technology and Engineering, University of the Sunshine Coast, Sunshine Coast, QLD, Australia; 3 School of Health, University of the Sunshine Coast, Sunshine Coast, QLD, Australia

**Keywords:** cell biology, *Chlamydia pecorum*, genomics, koala, *Phascolarctos cinereus*, review

## Abstract

*Chlamydia pecorum* is globally recognised as an important pathogen of both agricultural and conservation concerns, contributing to significant production losses in livestock and debilitating disease in koalas (*Phascolarctos cinereus*). This scoping review evaluates the current *C. pecorum* research trends and identifies critical knowledge gaps. A systematic literature search across six major databases yielded 2,099 records. Following screening and eligibility assessment using the PRISMA-ScR framework, supplemented with the Joanna Briggs Institute Manual for Evidence Synthesis: Scoping Reviews, 194 studies were included and categorised into seven thematic areas: diagnostics and surveillance (*n* = 56), genotyping (*n* = 32), genomics (*n* = 14), co-infections (*n* = 28), vaccines and therapeutics (*n* = 39), cell biology (*n* = 14), and literature reviews (*n* = 11). While the literature is dominated by studies on diagnostics and molecular epidemiology of livestock and koala infections, *C. pecorum* genomic data remain sparse, with only seven complete genomes available. The significance of *C. pecorum* infections in non-koala marsupials, wildlife and other domestic animals remains unclear. Similarly, the contributions of bacterial, viral, and protozoal co-pathogens to disease outcomes across hosts are still poorly defined. Functional cell biology studies are similarly underrepresented and rely on a narrow range of livestock and koala strains. Vaccine trials in koalas and sheep, while promising, have shown limited protection, with current formulations failing to achieve sterilising immunity. Overall, this review highlights the substantial progress in diagnostic and surveillance research while emphasising the urgent need for expanded genomic resources, broader isolate biobanks, and integrated, multidisciplinary approaches, to advance our understanding of *C. pecorum* cell biology, evolution, transmission, and host–pathogen interactions.

## 1 Introduction

The family *Chlamydiaceae* comprises a group of obligate intracellular Gram-negative bacteria responsible for a broad spectrum of diseases affecting humans, livestock, pets, and wildlife worldwide (1, 2). Among these, *Chlamydia abortus*, *C. psittaci*, *C. suis*, and *C. pecorum*, are particularly significant due to their impact on animal health, wildlife conservation, potential for zoonotic transmission, and economic consequences in livestock industries (3–9) Chlamydial infections in animals can be acute or chronic, but they often remain subclinical (2). This subclinical carriage may enable persistent environmental contamination and poses challenges for disease control (2).

*C. pecorum* is increasingly recognised as a multi-host pathogen with global distribution and diverse clinical manifestations (1, 2, 10–12). It infects both domestic and wild animals. In livestock (cattle, sheep, goats, pigs and water buffaloes), *C. pecorum* is associated with asymptomatic shedding as well as polyarthritis, encephalomyelitis, pneumonia, mastitis, enteritis, conjunctivitis, and reproductive failure, including fetal loss and infertility, all of which contribute to considerable production losses and animal welfare concerns (6, 10–12). Severe outcomes, such as fetal loss, are well documented in sheep, cattle, goats, and pigs (13–21). The presence of subclinical and persistent infections enables undetected transmission within and between herds, complicating disease management and surveillance efforts (11, 22–24).

*C. pecorum* has been detected in a range of wildlife species globally, including urban pigeons (25, 26), psittacine birds across Latin America, Australia and other regions (27–30), and wild ruminants such as alpine ibex, chamois, and red deer (31–33). However, *C. pecorum* is best known for being the main pathogen of koalas (*Phascolarctos cinereus*) across Australia, causing widespread infections and debilitating ocular and urogenital disease that contribute significantly to population decline (34, 35). Infected koalas may develop blindness, respiratory disease, cystitis, and infertility (36–41). Concurrent infection of *C. pecorum*, koala retrovirus (KoRV) and Phascolarctid gammaherpesviruses (PcGHV1 and 2) have been suggested to worsen disease outcomes (42–46). Despite intensive management, including antibiotic therapy and vaccination, the survival rate of treated koalas remains low, emphasising the need for improved understanding and intervention strategies (47). Given the high infection prevalence in koalas (37), the detection of *C. pecorum* in other marsupial species is not unexpected. Since the early 2000s, *C. pecorum* has been detected in several marsupial species, including gliders, possums, bandicoots, and quolls (12, 48, 49).

The broad host range, ubiquity, and pathogenic potential of *C. pecorum* suggest it is an emerging pathogen of considerable concern (11, 50). Molecular studies also suggest high genetic diversity of *C. pecorum* strains which may contribute to host adaptation (51). Nevertheless, much of the current research on *C. pecorum* remains heavily focused on gene-centric molecular epidemiology and strain-level characterization, with relatively few (seven) complete genome sequences available to support in-depth comparative and functional analyses (52, 53). Vaccine development efforts have advanced significantly, particularly in koalas, where immunization is considered a promising tool for conservation management (54). Recently, a vaccine developed at the University of the Sunshine Coast, Australia received Minor Use approval from the Australian Pesticides and Veterinary Medicines Authority (APVMA), showing to limit disease progression and reduce chlamydial-related mortality in koalas (55).

Given the global distribution, economic impact and ecological significance of *C. pecorum*, there is a compelling need to re-evaluate the current trends in *C. pecorum* research. This includes identifying existing knowledge gaps and emerging priorities, which are essential to inform integrated strategies for diagnosis, treatment, control, and prevention across its diverse host range. For this purpose, we conducted a scoping review to provide a comprehensive overview of the field and highlight critical avenues for future investigation.

## 2 Materials and methods

### 2.1 Protocol

To examine current research trends and the existing body of literature on *C. pecorum*, we conducted a scoping review following the expanded framework of the PRISMA for scoping reviews (56), supplemented with the Joanna Briggs Institute Manual for Evidence Synthesis: Scoping Reviews (57). Results were reported according to the PRISMA-ScR guidelines.

The five broad stages of this scoping review are as follows:

Stage 1: identifying research questions.

Our research question was “What are the current research trends and study types in the global literature on *C. pecorum* in animals?” and “Which areas of research require attention to improve the understanding of *C. pecorum*?”

Stage 2 and 3: identify and select relevant studies.

A comprehensive literature search was conducted across multiple databases - Google Scholar, EBSCO, Scopus, PubMed, Crossref, and Web of Science - using a set of predefined search terms (Table 1). Title and abstract screening were initially performed by two independent reviewers (Huong Q. Duong and Martina Jelocnik), followed by a full-text assessment to determine eligibility based on the inclusion criteria.

Stage 4: charting the data.

**Table 1:** Scoping review search criteria with search terms, databases, and exclusion criteria.

Scoping review concepts	Concept 1: pathogen	Concept 2: host	Concept 3: language and time frame	Concept 4: exclusion of search terms
Search terms	((“*Chlamydia pecorum*” OR *C. pecorum* OR *C. pec OR Chlamydophila pecorum*))	(animal* OR koala* OR livestock OR cow OR bovine OR swine OR pig OR sheep OR ovine OR goat OR ruminant* OR mouse OR wildlife OR “wild animal*”)	English, from 2010 to 2025, full text availability	Studies are not published in English, not in the 2010–2025 period, studies not involving *C. pecorum*.
Databases	Pubmed, Scopus, EBSCO, Google Scholar, Crossref, Web of Science			

The data collected in eligible studies were charted in Microsoft Excel spreadsheets. Charted data included authors, year of publication, title, study type, population, focus and results of the studies.

Stage 5: collating, summarising, and reporting the results.

Eligible studies were analysed for results specific to our research questions and implications for future research and practice.

### 2.2 Eligibility criteria

The PCC approach (Population, Concept, Context) [3] was used to examine the studies for eligibility, as described here.

#### 2.2.1 Population

The focus of this review is on animals affected by *C. pecorum*, including livestock (e.g., sheep, cattle, pigs, goats, buffaloes), koalas (*Phascolarctos cinereus*), and other free-ranging or captive wildlife species. These hosts are known to be susceptible to *C. pecorum* infection, which can result in a variety of clinical outcomes, ranging from subclinical shedding to significant disease manifestations.

#### 2.2.2 Concept

The central aim of this review is to identify and characterize the types of studies that have been conducted on *C. pecorum*. This includes research focused on diagnosis (molecular, serology, histology, immunoassay, and mixed methods) and surveillance (detection or prevalence of *C. pecorum*), genotyping (molecular characterization of the strains using *omp*A, MLST, other loci typing), genomics (molecular characterization of the strains using WGS), cell biology (cell cycle development, morphology), vaccine and vaccine development (trials) and other treatments, co-infection (concurrent infection of *C. pecorum* and other infectious agents) and reviews (summary of current literature). By examining the distribution of these study types over time and across species, the review provides a comprehensive overview of research trends, highlights shift in scientific focus and informs future directions for *C. pecorum* research.

#### 2.2.3 Context

The review was conducted from a global perspective, encompassing research undertaken in both veterinary and disease management settings. Given *C. pecorum*’s broad host range, potential for cross-species transmission, and emerging significance as a pathogen of ecological and economic concern, a global and cross-disciplinary approach is essential to fully capture the scope of research activity in this field.

### 2.3 Information sources

Online databases were used to identify publications between 2010 and 2025. Google Scholar, EBSCO, Scopus, PubMed, Crossref, and Web of Science were searched until the 17th of May 2025.

### 2.4 Search strategies

Eligible studies were identified using the above-mentioned databases. Filters were applied to restrict publication date, publication status and language. Results from databases were collaged and duplicates were removed using Paperpile reference software. Retraction and errata were checked.

### 2.5 Selection of sources of evidence

To ensure consistency in the decision-making process, both reviewers (HQD and MJ) screened the 194 publications, discussed the eligibility criteria, and evaluated the titles, abstracts, and full texts of all studies.

### 2.6 Data charting process

Data from eligible studies were extracted and collated according to key study characteristics. The reviewers discussed the charted results as a team and discrepancies of individual decisions were resolved by re-examination of the objectives of the review and eligibility and exclusion criteria.

### 2.7 Synthesis of results

Studies were grouped by study types: diagnosis and surveillance, genotyping, vaccines and other treatments, genomics, cell biology, co-infection, and reviews (Supplementary Table 1). Consistent with scoping review methodology, no formal quality assessment was performed because the aim of this review was to map the breadth and nature of the available evidence.

## 3 Results

### 3.1 Selection of sources evidence

**Figure 1:**
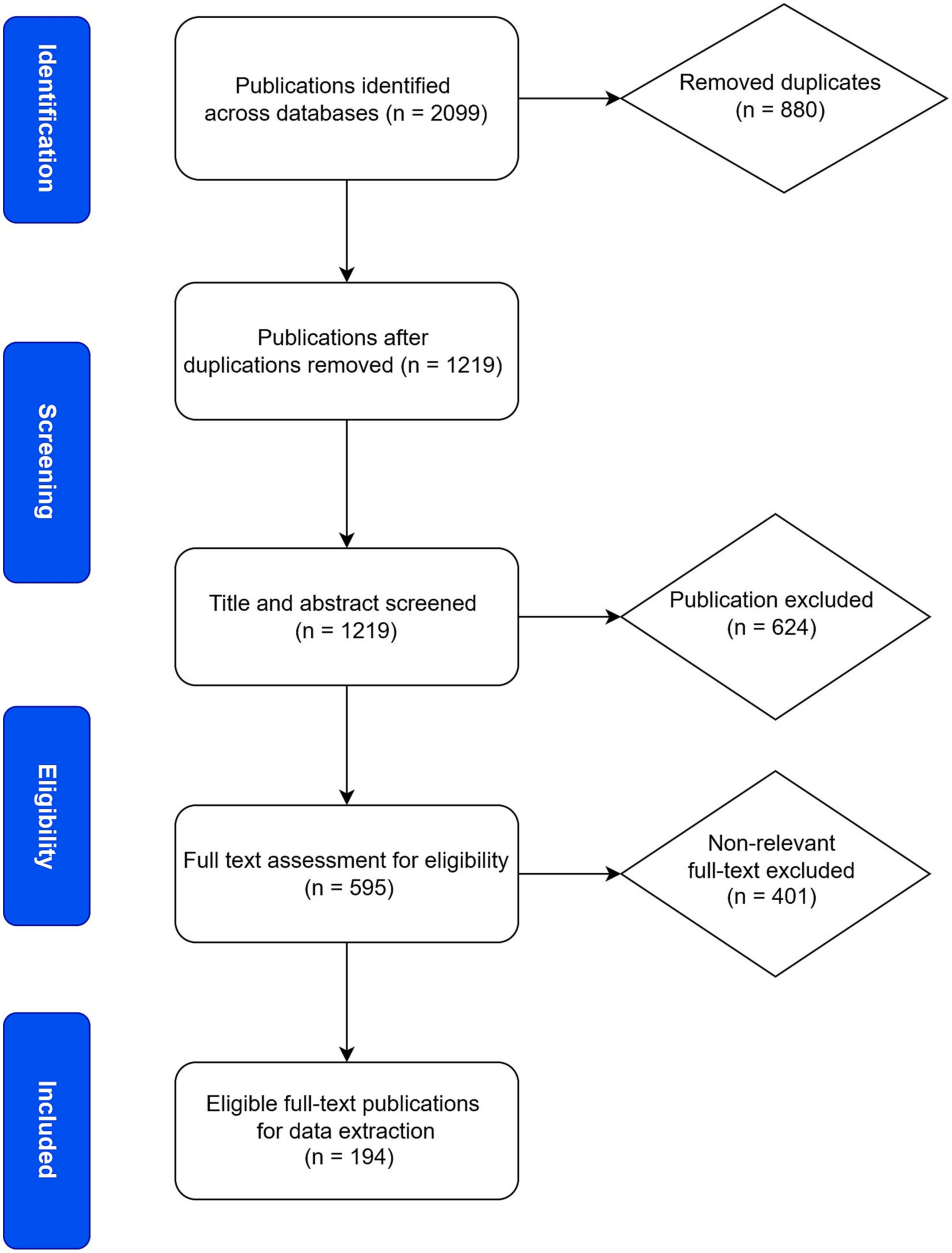
PRISMA flow chart for *C. pecorum* research scoping review. Through a literature search, 2099 records were identified, sourced from EBSCO (107 studies), PubMed (198 studies), Scopus (229 studies), Google Scholar (401 studies), Crossref (1,000 studies), and Web of Science (164 studies). 1,219 articles were identified after bibliography screening and removal of duplicates. These articles were screened by title and abstracts, and 624 articles were excluded based on the eligibility criteria (articles not in English between 2010 and 2025, not concerning *C. pecorum* research or involved in humans). As a result, 595 articles were subsequently assessed by full-text review, and 401 articles were removed due to non-relevancy. The final 194 articles were included for data extraction and charting.

A total of 2,099 records were retrieved from the designated electronic databases and imported into Paperpile reference management software (Version. 1.5.828). The records were sourced from EBSCO (107 studies), PubMed (198 studies), Scopus (229 studies), Google Scholar (401 studies), Crossref (1,000 studies), and Web of Science (164 studies). Following the removal of 880 duplicates, the remaining articles underwent title and abstract screening. During this stage, 624 articles were excluded based on predefined eligibility criteria, which included non-English language publications, lack of full-text availability, and studies not involving *C. pecorum*. The remaining 595 articles proceeded to full-text review, resulting in the exclusion of an additional 401 articles irrelevant to the scope of our research. As a result, 194 articles met the inclusion criteria and were selected for data extraction and charting. The detailed screening and selection process is illustrated in Figure 1.

### 3.2 Characteristics of sources of evidence

**Figure 2:**
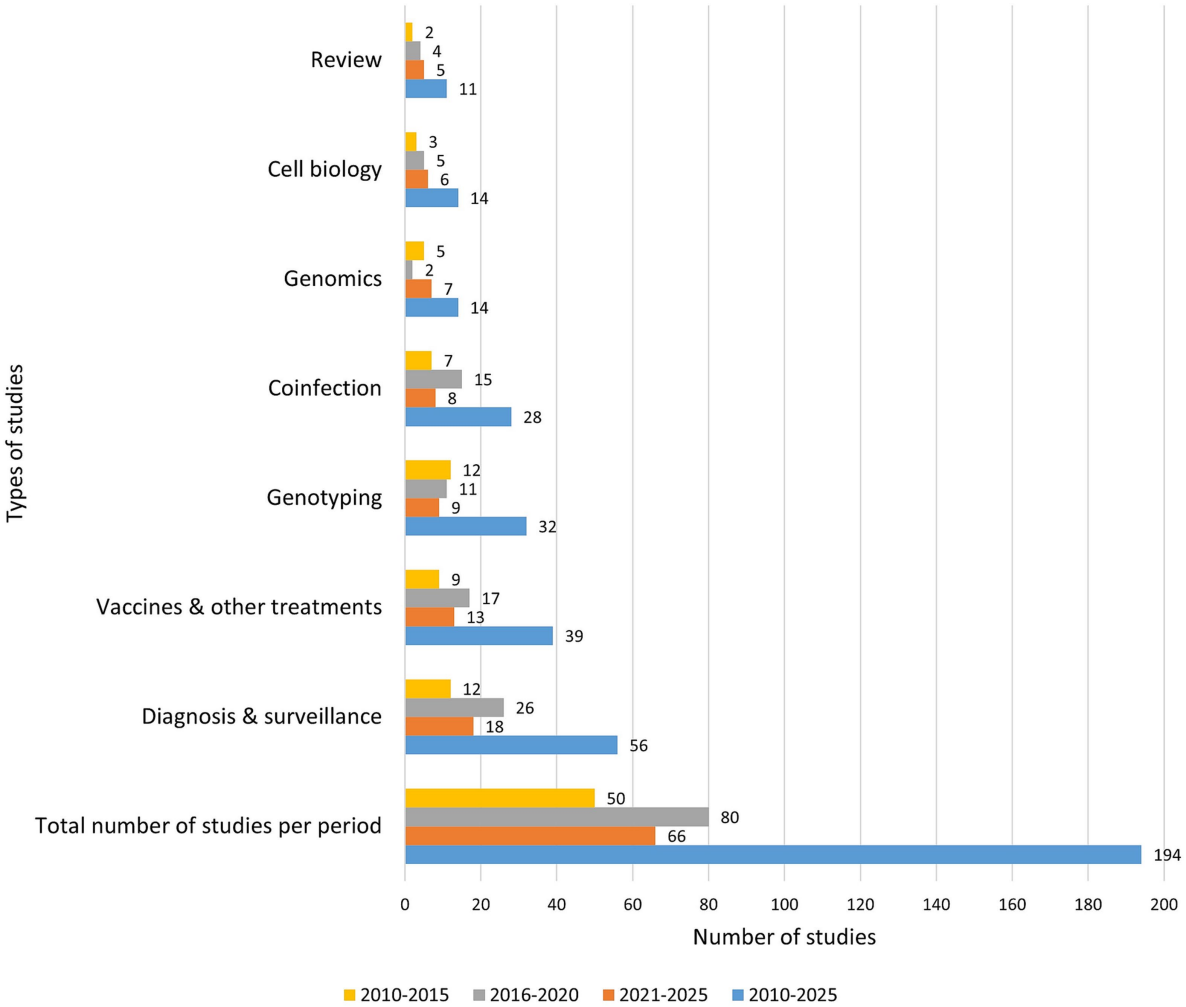
Research classification and distribution of identified studies over the 5-year periods (2010–2015; 2016–2020; 2021–2025). Study types are classified as either reviews (compilation of literature on *C. pecorum*); genomics (*C. pecorum* strain characterization by WGS); vaccines and other treatments vaccine trials and antibiotic treatments; diagnosis (studies that examine the diagnostic methods through molecular, serology, immunostaining, microarray, and histology) and surveillance (detection rate, prevalence, or distribution *C. pecorum* of in a population), and cell biology (the study of cell structure, function, development cycle, transformation) or co-infection (chlamydial and other agents infections).

A total of 194 relevant studies were classified into six major categories. Most publications (*n* = 56) focused on diagnosis and surveillance, followed by genotyping (*n* = 32) and coinfection studies (*n* = 28), highlighting these areas as the most extensively studied aspect of *C. pecorum* research in a range of hosts. Research on treatments including vaccines that explored both prophylactic and therapeutic interventions was also well represented (*n* = 39). In contrast, considerably fewer studies focused on cell biology (*n* = 14), and genomics (*n* = 14), indicating significant gaps in our understanding of the pathogen’s cellular mechanisms and genome biology. Additionally, literature reviews accounted for 11 publications, contributing to synthesis and contextualization of existing knowledge but underscoring the need for more primary research in underrepresented domains. Temporally, the number of *C. pecorum* studies was highest between 2016 and 2020 across almost all categories, except for the genomics category. These findings suggest that while diagnostic and treatment-oriented research is advancing, fundamental biological and genomic investigations require further attention (Figure 2).

### 3.3 Global distribution of *C. pecorum* studies and host range

**Figure 3:**
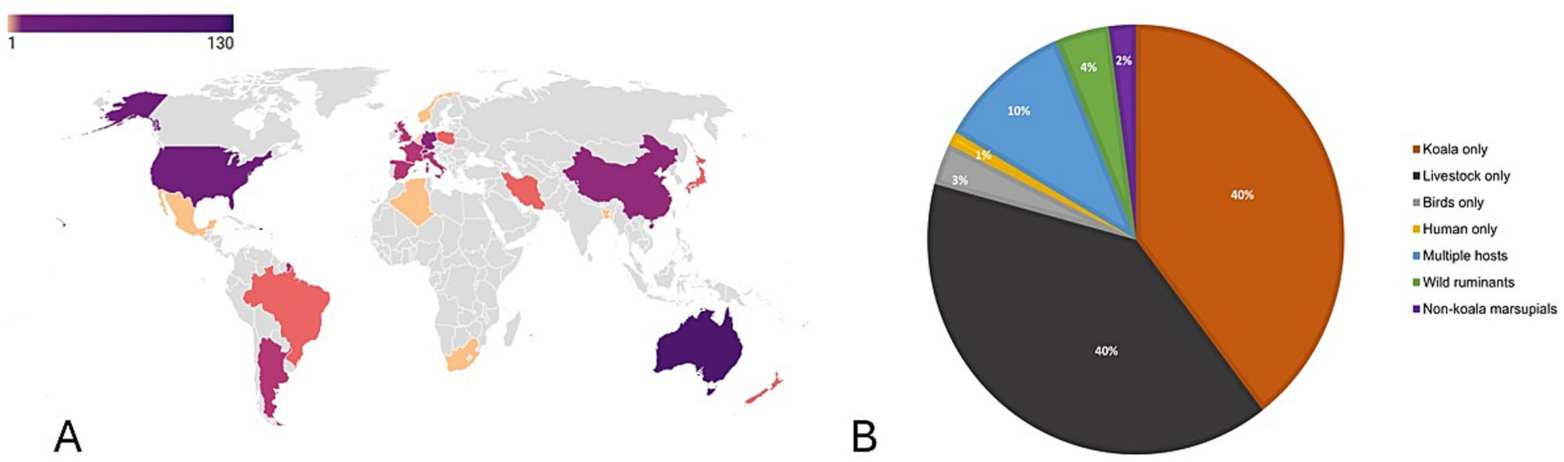
Global distribution of *C. pecorum* studies and host range (*n* = 194). **(A)** The majority of research studies cluster is in Australia (66.5%, *n* = 129) and Europe (18.5%, *n* = 36), followed by the Americas (8%, *n* = 16), Asia and South Pacific (6%, *n* = 11), and Africa (1%, *n* = 2). **(B)** The distribution of host range for *C. pecorum*. Most studies centered on koalas (40%) and livestock (40%). A small number (10%) focused on multiple hosts (mixed hosts of koalas, livestock, birds). Non-koala marsupials (2%) include southern greater sliders, bandicoots, quolls, and possums. Wild ruminants (4%) include red deer, reindeer, ibex, and chamois.

The results of this scoping review indicate predominant research is led in Australia (66.5%, *n* = 129), followed by Europe (18.5%, n studies = 36), the Americas (8%, n studies = 16), Asia and South Pacific (6%, n studies = 11), and Africa (1%, n studies = 2) (Figure 3A). Most studies were focused on livestock species (40%, pigs, cattle, sheep) and koalas (40%). Only 10% of studies included multiple host species (koalas, livestock, and birds). A smaller proportion of studies targeted non-koala marsupials (6%), birds only (3%), and humans (1%) (Figure 3B).

### 3.4 Methodological approaches and target genes

**Figure 4:**
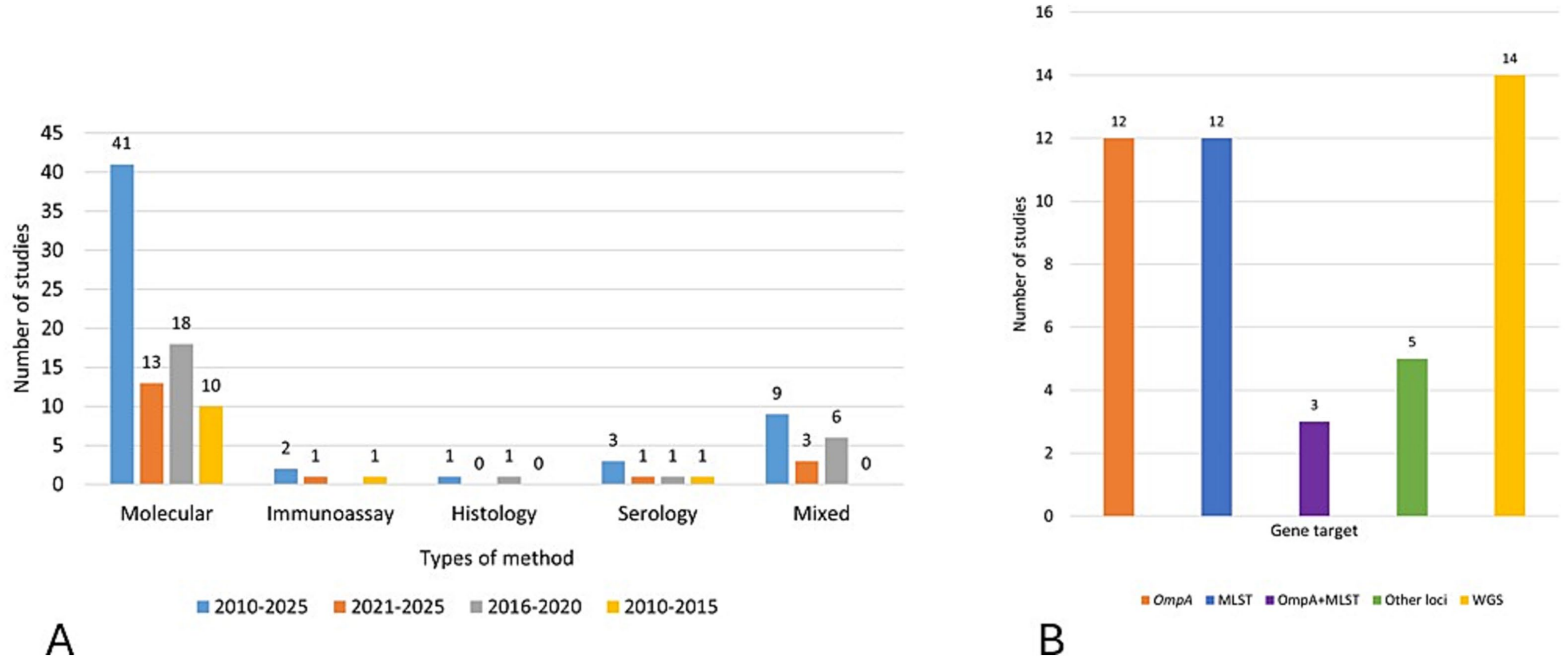
Diagnostic and molecular characterization methods used in *Chlamydia pecorum* studies (2010–2025). **(A)** Diagnostic methods of *C. pecorum* studies per 5-year periods (2010–2015; 2016–2021; 2021–2025). **(B)** Gene markers and WGS used in molecular characterization of *C. pecorum* strains.

A review of published studies on *C. pecorum* revealed clear disparities in the methodological approaches across the literature (Figure 4A). The overwhelming majority of studies focused on molecular diagnosis and speciation, accounting for 41 studies, with a slight increase in such studies in the 2016–2020 period. These investigations predominantly utilized PCR-based methods, including conventional PCR, and qPCR (including *C. pecorum*-specific assays). Among these, for detection and/or speciation, 16S rRNA was the most frequently targeted gene (21 studies), followed by the 23S rRNA gene (eight studies). Less frequently used targets for detection included the *C. pecorum*-specific *omp*B gene reported in seven studies and the hypothetical protein (HP) gene reported in five studies.

Other diagnostic methodological categories were less frequently represented. Serological investigations were employed in three studies, using complement fixation tests (CFT), enzyme-linked immunosorbent assay (ELISA), or peptide microarrays to evaluate host immune responses and were consistently employed throughout the span of the 15-year period. Immunoassay techniques, including immunofluorescence and immunohistochemistry, were observed in two studies, often used to confirm intracellular inclusions and tissue tropism. Histopathological studies were comparatively rare (one studies) typically restricted to case reports or post-mortem investigations in livestock and wildlife There are nine studies that utilized mixed methods (a combination of molecular and/or immunoassay, histology, and serology) (Figure 4A).

By contrast, *C. pecorum* genotyping studies represented a smaller but substantial proportion, with 32 studies. These studies were mainly gene-centric, often relying on markers such as *omp*A or multilocus sequence typing (MLST), with relatively few applying WGS approaches. The full or partial length *omp*A gene was used in 12 studies. In contrast, MLST as a primary method was applied in 12 studies, while mixed methods of using both *omp*A and MLST accounts for three studies, and five other studies used other loci such as ORF633*, inc*A, *tar*P, *Omc*B, *cop*N, membrane attack complex/perforin MACPF, putative Type III effector protein or polymorphic membrane protein *pmp*G1. Whole genome sequencing was utilized in 14 studies (Figure 4B).

### 3.5 Co-infection studies

There are 28 studies that addressed the co-infection between *C. pecorum* and other infectious agents. However, in the last decade, most of these studies (*n* = 11) focused on co-infections of koala retrovirus (KoRV) and chlamydia in koala hosts (42, 46, 58–67). In addition to KoRV, Phascolarctid gammaherpesviruses (PcGHV1 and 2) have also been detected in koalas with concurrent chlamydial infection, particularly in older or reproductively compromised koalas with urogenital pathology (*n* = 4) (42–46). In one study investigating transcriptomes of 26 koalas with ocular chlamydial infection, Burpengary virus, a novel picorna-like virus was also detected (68).

In farmed livestock and farmed foxes, mixed infections of *C. pecorum* with porcine epidemic diarrhea virus (PEDV), and related chlamydial species *C. abortus*, and *C. suis* were recorded. Prevalence of co-infection with *C. abortus* can be as high as 25% in sheep (18, 69) and 63% in farmed foxes (70), and 12.5% with *C. suis* in pigs (71, 72). Bacterial co-pathogens such as *C. suis* have also been detected with *C. pecorum* in ruminants and pigs (72). In wild birds, mixed infections with beak and feather disease virus (BFDV) were also readily detected, with up to 38.9% detection rate (27).

### 3.6 Genomes studies

In total, there are only seven *C. pecorum* complete genomes available, two derived from koala isolates (MarsBar_2018 and DBDeUG_2018) (53) and five from livestock strains (cattle E58, PV3056/3, NSW/Bov/SBE; and sheep W73, P787) (48, 53). In addition, 12 draft genomes have been published, and of those, three are from koalas, (SA/k2/UGT; Gun/koa1/Ure, IPTaLE), three from pigs (L1, L17, L71); four from sheep (IPA, M5, M6, M18), one from water buffalo (PV6959); and one from chamois (PV7855) (12, 19, 48, 51, 73–75). These 14 studies mainly focused on the *C. pecorum* genome structure and phylogenetic relationships between the strains, with some also including genotyping.

### 3.7 Functional cell biology studies

There are 14 studies that have addressed aspects of *C. pecorum* cell biology, and among these, the majority have focused on koala (Marsbar_2018) (76), sheep (IPA, W73, JP-1-751), cattle (E58), and/or pig (1710S) isolates (71, 77–86), highlighting a narrow strain diversity and biobank in the existing literature. Notably, just six studies have examined *C. pecorum* specifically (10, 76–78, 81, 82), and of these, only five involved culture-based approaches (76–78, 81, 82).

### 3.8 Antimicrobial and vaccine studies

There are 13 vaccine trials for *C. pecorum*, 11 in koalas and two in sheep, of which 12 studies targeted protein and one targeted whole cell (54). These trials tested the safety and efficacy of candidate vaccines. The majority of studies have focused on target the major outer membrane protein (MOMP) (87–98), sometimes in combination with other antigens such as NrdB, omp85a, and PmpG, and were tested with a range of adjuvants, including ISC, Alhydrogel, TiterMax, and Tri-Adjuvant (54). In parallel, the immunological response to vaccines has also been explored in 15 studies (87, 89, 90, 93–95, 97, 99–106). Notably, most of these studies have focused on koalas as the host species, whereas only two studies have addressed immune responses in sheep (97, 103).

A total of 12 studies has investigated the treatment of *C. pecorum* using antibiotics, primarily assessing therapeutic efficacy and comparative effectiveness of these antimicrobial agents (47, 107–115). These agents are chloramphenicol, florfenicol, enrofloxacin, azithromycin, and doxycycline. The use of peptide cathelicidin PhciCath5, and a HtrA serine protease inhibitor JOI146 are still under development (116, 117).

## 4 Discussion

To date, research on *C. pecorum* has largely centered on livestock and koalas, with limited investigation in other marsupials, ruminant wildlife, birds, or humans. Molecular work still relies heavily on conserved markers such as *omp*A, while studies on co-infection, genomic, and functional cell biology remain sparse. This constrained focus leaves critical gaps in our understanding of the pathogen’s true host range, cross-species transmission risks, functional biology and genomic data, highlighting the urgent need for broader and integrative investigations.

### 4.1 Diagnostics and epidemiology of *C. pecorum*: innovation or reinvention?

Globally, in the past decade, molecular detection of *C. pecorum* using species-specific qPCR has revealed both expected and unexpected hosts, confirming the mainstay of molecular diagnostics as well as ubiquitous prevalence of this species (2, 30, 33, 118–121). The overall prevalence of *C. pecorum* has been reported at approximately 30% in livestock and ranges from 20% to as high as 80% in some koala populations (11, 61, 122). Serological assays have also been widely employed to detect anti-*Chlamydia* antibodies however they are associated with cross-reactivity to other chlamydial species, have lower sensitivity and are therefore not the preferred diagnostic tool (6, 20, 69, 121, 123–131).

While qPCR continues to be the principal method for detecting chlamydial infections of animals and humans in the research as well as diagnostic laboratories (118, 132–134), more rapid and portable isothermal assays have been developed for *C. pneumoniae*, *C. psittaci*, *C. trachomatis* and *C. pecorum* (133–137). Currently, there are no widely available commercial point-of-care molecular assays that enable rapid and decentralized diagnosis of chlamydial infections. This persistence on centralized molecular testing highlights the need to enhance diagnostic capabilities across the broader Chlamydia research field.

### 4.2 Molecular epidemiology of *C. pecorum*: on a quest of uncovering the true genetic diversity

Efforts to explore *C. pecorum* strain diversity have historically adopted a gene-centric approach, targeting established genetic markers such as *omp*A, or in some studies, additional genes including *inc*A, ORF663, and *tar*P (33, 37, 122, 123, 138–140). The reliance on *omp*A, which encodes the major outer membrane protein MOMP, has provided a convenient and accessible marker but is not without limitations. Most studies sequence only short *omp*A fragments, despite the gene’s high polymorphisms and frequent recombination, both of which obscure true phylogenetic relationships and limit its reliability in cross-species comparisons (122). Studies utilizing *omp*A genotyping of both koala and livestock strains have revealed both highly clonal and highly diverse genotypes (12, 141, 142). Consequently, phylogenies based solely on *omp*A have shown poor congruence with those derived from other gene targets or WGS, raising concerns about its validity for strain differentiation and evolutionary inference. Moreover, *omp*A lacks reliability when applied to cross-species comparisons within the *Chlamydia* genus, further questioning its use in broader molecular epidemiological studies (122).

In response to these shortcomings, more centralized *C. pecorum* MLST scheme have been developed in 2013 to improve strain discrimination and evolutionary inference (15, 31, 143). *C. pecorum* MLST studies provided new insights into genetic diversity, at times conflicting with that of *omp*A. *C. pecorum* MLSTs showed clonal lineages, such as found in livestock only and those denoted ST23 and highly conserved *omp*A; less diverse STs where *omp*A and, more diverse ST where *omp*A is of the same genotype, as seen in koala strains (143). However, the resolution of MLST also remains constrained compared to whole-genome approaches.

### 4.3 Genomic data deficit for *C. pecorum*: implications for molecular epidemiology and comparative genomics

Despite substantial advances in culture-independent WGS approaches, such as probe-capture techniques successfully applied to *C. trachomatis* and *C. psittaci*, progress in generating high-quality, complete *C. pecorum* genomes has been minimal over the past 15 years (144–148). In contrast, *C. trachomatis* currently has almost 400 complete genomes of over 9,000 genome data (149), while *C. psittaci* is represented by over 100 genomes, of which at least 32 are complete (150, 151). These resources have allowed detailed genomic and functional studies, providing greater insights into virulence, host adaptation, and evolutionary dynamics - an opportunity that remains largely inaccessible for *C. pecorum.*

To date, only a handful of complete genomes (seven) and a small number of draft assemblies (12) have been published, representing isolates from koalas, sheep, cattle, pigs, water buffalo and chamois (12, 19, 48, 51, 53, 73–75). These genomes provided critical insights into phylogenetics and comparative genomics, identifying gene content with metabolic functions, polymorphic membrane proteins (Pmps), type III secretion effectors (T3SS), plasticity zone (PZ), and a chlamydial plasmid (12, 53, 73). More importantly, many *C. pecorum* draft assemblies lack essential regions commonly associated with virulence, or they contain poorly resolved genomic segments, which significantly limit their utility for detailed comparative analyses (53). Consequently, investigations into the origin, strain diversity, and potential virulence determinants of *C. pecorum* infections particularly in koalas remain heavily constrained by the paucity of available genomes. This limitation contrasts sharply with recent genomic studies of *C. psittaci*, which were able to demonstrate that equine and human infections resulted from spillover events originating from psittacine hosts (147, 148), highlighting the power of comprehensive genomic datasets to resolve transmission pathways. Expanded access to complete genomes is essential to refine molecular epidemiology, understand host adaptation and genetic basis of *C. pecorum* pathogenicity. However, due to the high cost associated with WGS, particularly when involving probe-based approaches, *omp*A and MLST still remain valuable molecular tools for assessing genetic diversity.

### 4.4 Expanding the host range: a critical gap in *C. pecorum* research

Research on *C. pecorum* has primarily centered on two major host groups: koalas and livestock species, particularly sheep and cattle. While these investigations have substantially advanced our understanding of disease pathology, transmission, and molecular diversity within these hosts, there remains a significant knowledge gap regarding the prevalence, and impact of *C. pecorum* across the wider spectrum of potential hosts.

In Australian marsupials, for example, *C. pecorum* has been detected in several species beyond koalas, including gliders, possums, bandicoots, and quolls (49). Despite this, the clinical significance of infection in these hosts remains unresolved, and there are still no confirmed reports of infection in kangaroos or wombats. The absence of data in these taxa may reflect true host resistance but is equally likely a product of limited surveillance. Similarly, *C. pecorum* DNA has been detected globally in various wild ungulates, including red deer, alpine ibex, and chamois (26, 31, 32, 52), highlighting its presence in free-ranging ruminants. These findings emphasize the need to expand wildlife surveillance to determine whether these species act as incidental hosts or potential reservoirs.

In domestic animals, *C. pecorum* is well recognised in sheep, goats, pigs, and cattle, yet its presence in other domestic species (e.g., horses, poultry) remains poorly defined (10, 11, 16, 18). Habitat overlap further complicates the disease landscape. In Europe, for instance, *C. pecorum* has been identified in both livestock and wild ruminants that share grazing areas (19, 32, 33), as well as in birds across the world (25, 26, 28–30) raising the likelihood of cross-host transmission. Similar risks exist in Australia where marsupials, livestock, and birds frequently share overlapping habitats. Such ecological contexts provide opportunities for *C. pecorum* to move across host boundaries, with poorly understood consequences for disease dynamics. Finally, one recent study described detection of *C. pecorum* DNA in a bronchoalveolar lavage fluid from a man presenting with severe community-acquired pneumonia and respiratory failure, potentially acquired as zoonotic infection (8). While zoonotic potential of *C. pecorum* is perhaps not surprising due to recognized zoonotic potential of related chlamydiae (5, 9, 27, 152), human *C. pecorum* infection is scarce (8).

Integrated, multi-host surveillance frameworks, coupling molecular epidemiology with longitudinal studies of infection outcomes will be essential to clarify host susceptibility, identify potential reservoirs, and better define the ecological network of *C. pecorum* transmission.

### 4.5 The underexplored role of co-infections in *C. pecorum* pathogenesis

One of the most overlooked areas in *C. pecorum* research is the role of co-infections in disease outcomes. Historically, most studies have focused almost exclusively on *C. pecorum* as a single-agent, largely neglecting the potential influence of co-infecting pathogens (66). Growing evidence shows that viral, bacterial and even protist co-infection may significantly alter the pathogenesis and persistence of *C. pecorum.* In koalas, several studies have suggested a synergistic interaction between *C. pecorum* and KoRV, where retroviral-induced immunosuppression may exacerbate the severity or chronicity of chlamydial disease (52, 64, 122, 153). Associations between *C. pecorum* and PcGHV1 and 2 are also strongly suggested, given their high prevalence and immunosuppressive properties, with possible links to more severe disease outcomes (48, 52, 59). Similarly, in pigs, co-infection with PEDV has been shown to induce *C. pecorum* persistence *in vitro*, heightening the potential for viral co-infections to alter chlamydial developmental cycles (71). Despite these observations, the clinical significance and mechanistic basis of these viral interactions remain poorly understood, and systematic studies are lacking (154–156).

Beyond viral interactions, bacterial co-infections are increasingly recognized as important modifiers of *C. pecorum* pathogenesis. In small domestic ruminants, *C. pecorum* was associated with infectious keratoconjunctivitis in the presence of *Mycoplasma conjunctivae*, indicating a cooperative interaction that exacerbates clinical disease (16). Co-infection with *C. abortus* and *Coxiella burnetii* has also been reported in ruminants, with direct links to abortion (17, 157). In a recent study, co-infection with *Bordetella bronchiseptica* and *C. pecorum* was commonly observed in koalas and was associated with severe respiratory disease; however, whether a synergistic interaction exists between these pathogens remains to be determined (158).

Collectively, the lack of co-infection studies represents a major limitation in our understanding of disease ecology. Future research must adopt a multi-pathogen approach, to better capture the complexity of infectious disease and interaction dynamics in these animals.

### 4.6 Limited functional cell biology studies on *C. pecorum*

In *C. pecorum* research, there remains a significant deficit in our understanding of its functional cell biology. This limited body of work highlights a broader research gap: the lack of diverse, well-characterised *C. pecorum* strains beyond koalas and livestock strains for experimental use (71, 76–78, 82, 84). Without expanding the range of available isolates in biobanks, our ability to model disease processes and develop targeted interventions remains severely constrained (77, 147).

To successfully complete their developmental cycle and manipulate host cellular processes, *Chlamydia* spp. secrete numerous effector proteins such as SINC and Tarp, into the host cell cytoplasm (159, 160). Previous studies have shown that T3SS effectors such as SINC can localise to the nuclear envelope of both infected and neighbouring uninfected cells and interact with host proteins that regulate nuclear structure, chromatin organisation, gene silencing and signalling (161). Similarly, Tarp has also been identified to manipulate host-actin cytoskeleton in early stages of *C. trachomatis* infection (162). Gene homologues of SINC and Tarp have been identified in *C. pecorum* genomes (53) but their functions remain uncharacterised.

*C. pecorum* also harbours toxin genes (*tox*A, and *tox*B) which may function as secreted effectors although experimental confirmation is lacking (53). Plasmid-associated regulation of chromosomal genes involved in metabolism and inclusion biology as well as the highly polymorphic membrane proteins Pmps are also thought to contribute to host invasion and pathogenesis but their precise role in *C. pecorum* biology remain poorly defined (73, 141, 163). The development of species-specific shuttle vector transformation systems for *C. pecorum* enabled stable genetic modification and fluorescent tracking of infection, providing essential tools for investigating virulence, plasmid biology, and intracellular development in these medically and veterinary important pathogens (142). A critical next step is to apply functional cell biology approaches to elucidate how *C. pecorum* operates within host cells, as genomic predictions alone cannot resolve pathogenesis.

### 4.7 From antibiotics to vaccines: progress and pitfalls in combatting *C. pecorum*

Vaccine research in *Chlamydia* has advanced considerably, with over 220 trials in the last seven decades, encompassing different chlamydial species, formulations and delivery platforms. *C. trachomatis, C. muridarum* and *C. psittaci* dominate the field with 84, 78 and 26 trials, respectively, (54). *C. pecorum* vaccine development is not lagging. Most koala vaccines, based on recombinant MOMP (MOMP A, F and G) subunit formulations, provide broad antigenic coverage (54, 164, 165). The use of adjuvants such as Tri-Adjuvant has enhanced immune responses and enabled single-dose administration (92–94, 101). Several vaccines have been shown to induce both humoral and cell-mediated immune responses, including neutralising antibodies and cytokine profiles (87, 88, 91, 94, 95, 97, 101). However, long-term protection and sterilising immunity remain unachieved (101). The magnitude of immune responses appeared to vary between individuals, and the presence of antibody or cytokine responses did not always consistently predict protection from infection or clinical disease. Differences in immune responsiveness have been linked to host factors such as infection status, immunogenetic variation (63, 92, 93, 97, 101), vaccine formulation and route of administration (89–95). In livestock, a live *C. abortus* vaccine offered partial cross-protection for *C. pecorum* in sheep but lacked species specificity (166) while recombinant PmpG and MOMP antigens elicited modest responses, indicating the need for multi-antigen approaches (97).

Treatment of *C. pecorum* relies on antimicrobials though outcomes are variable and reinfection is common (112). In koalas, antibiotics can severely disrupt their gut microbiome critical for eucalyptus digestion (110). Doxycycline is the most effective option, with cure rates of up to 97% (47, 112) while chloramphenicol and other agents offer only partial or inconsistent efficacy (47, 107, 108, 112, 113, 115). Although resistance genes for chloramphenicol and doxycycline have been detected in koala microbiomes, they have not been identified in *C. pecorum* (110). Emerging therapies such as the HtrA protease inhibitor (JO146) remain under development (117). Overall, these findings point to several unresolved questions crucial for improving therapeutic outcomes and controlling transmission across hosts.

This scoping review has several limitations. The search strategy was limited to selected electronic databases and English-language only publications, which may have resulted in the omission of relevant studies. As expected for a scoping review, no formal appraisal of methodological quality was undertaken, and therefore the findings reflect the scope rather than the strength of the available evidence. The included studies were highly heterogeneous with respect to host species, study design, and analytical methods, which limited direct comparisons across studies. Studies were eligible if they included data on *C. pecorum*, even when additional *Chlamydia* species were investigated, which may have introduced variability in the interpretation of species-specific outcomes. Furthermore, the existing literature is heavily weighted toward gene-targeted molecular epidemiology, which may bias the apparent distribution of research themes compared with genomics, cell biology, and functional studies.

## 5 Future directions and conclusions

To date, substantial gaps remain in our understanding of *C. pecorum* pathogenesis, host range, and phylogenomics. Research to date has focused predominantly on koalas and livestock, leaving the presence, prevalence, and clinical significance of *C. pecorum* in other domestic and wild species largely unresolved. Expanded, systematic surveillance across diverse taxa is essential to define the true host range, identify potential reservoirs, and evaluate cross-species transmission risks. Genomic resources for *C. pecorum* also remain strikingly limited. Generating more complete genomes, spanning diverse hosts and regions, will be critical for elucidating virulence determinants, and improving phylogenetic relationships between the strains. Similarly, functional studies have been constrained by the scarcity of well-characterised isolates suitable for experimental work. A broader and genetically diverse isolate biobank is urgently needed to investigate mechanisms of persistence, immune evasion, tissue specificity, and host adaptation, particularly given the distinct disease phenotypes observed across species. Likewise, the effects of co-infections remain poorly understood, despite growing evidence that viral, bacterial and protist co-pathogen may significantly modulate disease severity and pathogen dynamics.

Given the extensive overlap between wildlife, livestock, and human environments, *C. pecorum* research now requires a more integrated, One Health-aligned approach. Such a framework, combining genomics, functional biology, epidemiology, and ecosystem-level surveillance, will be essential to quantify transmission pathways, assessing emerging public health implications, and informing targeted control strategies if required. By expanding research beyond the traditional host systems and employing modern comparative and functional genomic tools, the field can advance towards improved disease management, conservation, and veterinary health across diverse species.

## Data Availability

The original contributions presented in the study are included in the article/Supplementary material, further inquiries can be directed to the corresponding author.
